# Anti-inflammatory Effects of GTE in Eye Diseases

**DOI:** 10.3389/fnut.2021.753955

**Published:** 2021-12-13

**Authors:** Jian Li, Lin Du, Jing Na He, Kai On Chu, Cosmos Liutao Guo, Mandy Oi Man Wong, Chi Pui Pang, Wai Kit Chu

**Affiliations:** ^1^Department of Ophthalmology, Affiliated Hangzhou First People's Hospital, Zhejiang University School of Medicine, Hangzhou, China; ^2^Department of Ophthalmology and Visual Sciences, The Chinese University of Hong Kong, Hong Kong, Hong Kong SAR, China; ^3^Bachelor of Medicine and Bachelor of Surgery Programme, The Chinese University of Hong Kong, Hong Kong, Hong Kong SAR, China; ^4^Hong Kong Eye Hospital, Hong Kong, Hong Kong SAR, China

**Keywords:** green tea, EGCG, ocular inflammation, pharmacokinetics, safety

## Abstract

Ocular inflammation is a common complication of various eye diseases with wide consequences from irritations to potentially sight-threatening complications. Green tea is a popular beverage throughout the world. One of the proven health benefits of consuming green tea extract (GTE) is anti-inflammation. Catechins are the biologically active constituents of GTE. In *in vitro* and *in vivo* studies, GTE and catechins present inhibition of inflammatory responses in the development of ocular inflammation including infectious, non-infectious or autoimmune, and oxidative-induced complications. Research on the ocular inflammation in animal models has made significant progress in the past decades and several key disease mechanisms have been identified. Here we review the experimental investigations on the effects of GTE and catechins on various ocular inflammation related diseases including glaucoma, age-related macular degeneration, uveitis and ocular surface inflammation. We also review the pharmacokinetics of GTE constituents and safety of green tea consumption. We discuss the insights and perspectives of these experimental results, which would be useful for future development of novel therapeutics in human.

## Introduction

### Ocular Inflammation

**Figure 1:**
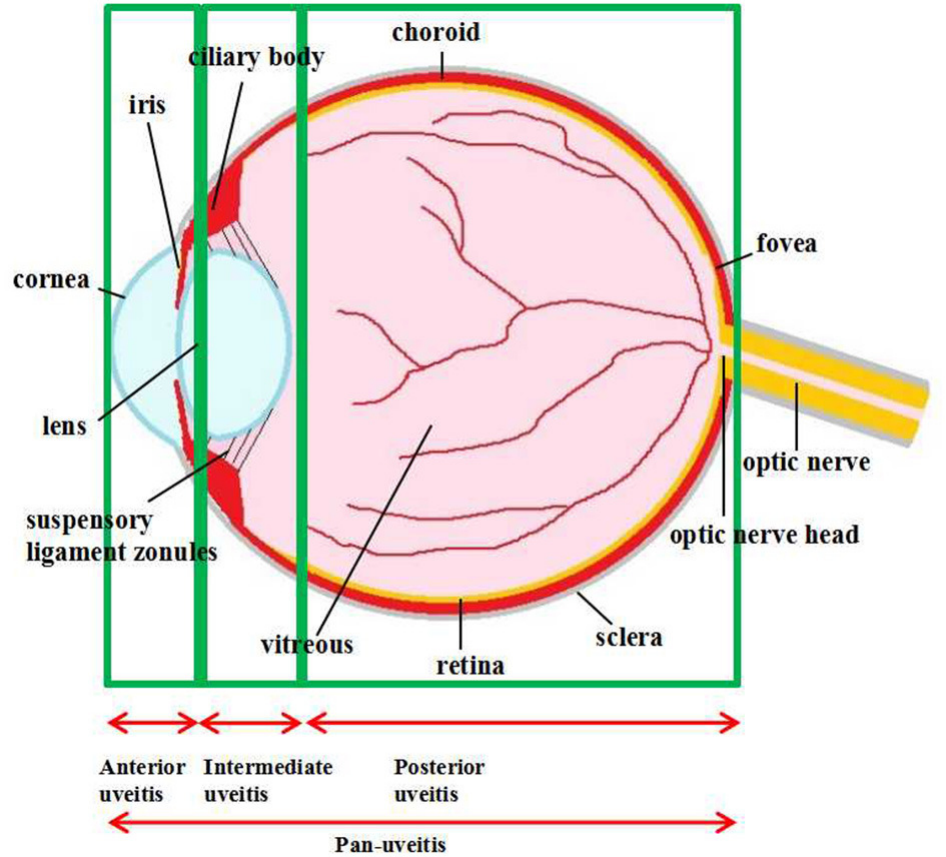
Anatomical classification of uveitis. Uveitis is classified as anterior, intermediate, posterior uveitis, and panuveitis according to the anatomic location of the inflammatory process.

Multiple eye tissues, from ocular surface to intraocular tissues, could be targeted by inflammation. One major entity of ocular inflammation appears in uvea, which leads to uveitis, a complex group of manifestations of intraocular inflammation with variable consequences ranging from persistent irritations, pain, and vision impairment. Uveitis is also a potentially blinding disease, which causes ~10-15% of visual impairment worldwide and 25% of legal blindness in developed countries (1, 2). Uveitis represents any intraocular inflammation from the uvea which comprises the iris, ciliary body, and choroid as well as the vitreous, retina, and even a part of optic nerve (3). Based on inflammatory involvement of the anatomic framework, the International Uveitis Study Group (IUSG) classified uveitis into anterior, intermediate, posterior, or pan-uveitis (4) (Figure 1). The prevalence and characteristics of various uveitis subtypes depend on age, gender, genetic background and geographic distribution (5, 6). Uveitis can be limited to the eye or associated with systemic syndromes, such as ankylosing spondylitis, Behçet disease, and Vogt–Koyanagi–Harada disease (7). Though the precise pathogenesis is complicated and still unclear, accumulating evidence has demonstrated that some uveitis sub-types are caused by infectious agents and could be rehabilitated by specific antimicrobial therapies with or without corticosteroids (8). Other forms of uveitis may be of autoimmune nature which the underlying infectious trigger could not be identified. Thus, for the autoimmune uveitis, corticosteroids are the mainstay of therapy (9). However, there are some uveitis patients who fail to respond to these treatments, or responses being inconsistent. In addition, these treatments may have several intraocular and systemic side effects such as high intraocular pressure and nephrotoxicity (10). In cases of severe sight-threatening uveitis, surgeries are needed to prevent the secondary visual impairments (11). Other therapies including antimetabolites, T-cell inhibitors, alkylating agents, and biologics, have been developed vastly in the past 40 years. However, none of these agents is able to represent the perfect sole treatment with each owning particular side effects (12). Research is still needed to improve the efficiency and safety of treatments against uveitis.

Apart from the uveitis, other ocular abnormalities can also be severely affected by inflammation. Glaucoma, a leading cause of blindness characterized by progressive loss of retinal ganglion cells (RGC) and the corresponding visual field, has been associated to ocular inflammation, especially for primary open angle glaucoma (POAG) and normal tension glaucoma (NTG) (13–15). In glaucomatous RGC, inflammatory cytokines (16) and pathways (17, 18), as well as HIF-α (19), were up-regulated in response to intraocular pressure (IOP) elevation, vascular dysfunction, and ischemia. IOP lowering is the major strategy for management of glaucoma. However, RGC death and visual field impairment are irreversible. Interestingly, recent studied identified a link between neuroinflammation and glaucoma (20). IOP elevation could activate retinal microglial cells, which could then induce the expression of proinflammatory cytokines including IL-6 and TNF-α (21). These studies highlighted inflammatory regulation could be another aspect for the treatment.

Age-related macular degeneration (AMD), the most common cause of irreversible vision impairment in aged population in developed countries, is characterized by retinal pigment epithelium (RPE) damage, extracellular drusen, death of photoreceptors, and neovascularization (22). AMD can be divided into dry and wet forms, also known as geographic atrophy and exudative AMD, respectively. Wet AMD could be manageable due to the application of anti-VEGF agents (23). Unfortunately, the treatment of dry AMD, which accounts for the majority of AMD cases, is still challenging (24, 25). Various local and systemic inflammatory molecules, such as NLRP3 inflammasome (26, 27), and circulating complement components (28–32), were activated in AMD, indicating inflammation is involved in the pathogenesis of AMD.

Ocular surface diseases are heterogeneous and many of them share a common characteristic of inflammation including dry eye. As defined in the Dry Eye Workshop (33), dry eye disease is a multifactorial disorder of the tears and ocular surface, associated with symptoms of discomfort, visual disturbance, and tear film instability. It is accompanied by increased osmolarity of the tear film and inflammation of the ocular surface. The prevalence of dry eye ranged from 5 to 50% (34). Apart from age, sex and race, other consistent risk factors were identified such as chronic inflammation related meibomian gland dysfunction and Demodex eyelid infestation, connective tissue disease, conditions that may worsen tear stability and ocular surface (34, 35). Sjögren's syndrome is a chronic and progressive autoimmune disease that would lead to lymphocyte infiltration into lacrimal glands which results in dry eye. Keratoconjunctivitis sicca is the most common ocular manifestation in Sjögren's syndrome (36). In addition, topical anti-glaucoma medication become an essential treatment in controlling glaucomatous progression by lowering IOP. However, the need for long-term use, the presence of preservatives and concomitant use of multiple classes of topical anti-glaucoma medication increase the risk of developing ocular surface disease (37). For example, benzalkonium chloride was found to cause conjunctival inflammation and epithelial toxicity (38). To treat these ocular surface diseases, many therapeutic targets in the inflammatory cascade have been identified for ocular surface inflammation. Topical use of cyclosporine (39) and lifitegrast (40) targeting the inflammatory components are available in clinical practice to manage dry eye.

### Green Tea Catechins

**Figure 2:**
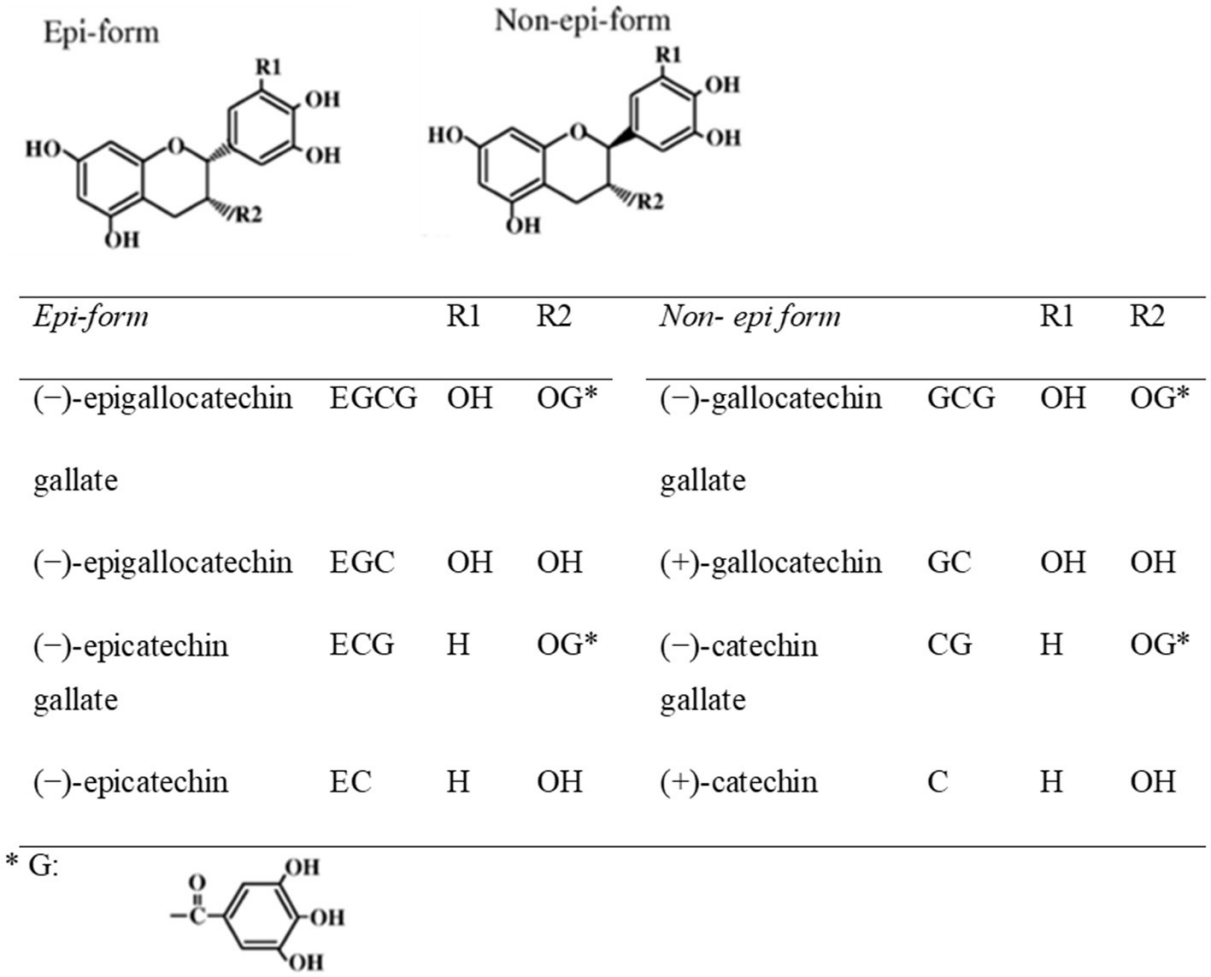
Chemical structures of eight green tea catechins (45).

Teas can be categorized into non-fermented green tea, semi-fermented oolong tea, and fermented black tea, according to various manufacturing processes. Green tea is the non-fermented leaves of plant *Camellia Sinensis*. Green tea preserves the natural structure of green tea polyphenolic constituents due to the initial stages of manufacturing. Green tea polyphenols include catechins, anthocyanins, flavones, and phenolic acids. To obtain green tea extract (GTE), fresh leaves have to go through complicated manipulations in laborious and well controlled extraction procedures. In brief, catechins are usually purified from green tea leaves by extraction with water and ethyl acetate, followed by chromatography using water/alcohol to elute the catechins (41). Components of tea leaves include: (1) tea polyphenols (tea catechins) (20%), (2) saccharides (including sucrose and glucose) (6%), (3) minerals (including potassium, magnesium, calcium, and aluminum) (5%), (4) amino acids (including Theanine and Glutamic acid) (4%), (5) caffeine (3%) and (6) insoluble substances (including polysaccharides, proteins, and pigments) (62%). The major constituents of green tea extract (GTE), derived from the leaves, include (–)-epigallocatechin gallate (EGCG) (>65%), (–)-epicatechin (EC) (<10%), (–)-epigallocatechin (EGC) (<10%), (–)-epicatechin gallate (ECG) (<10%), (+)-catechin (C), (+)-gallocatechin (GC), (–)-catechin gallate (CG) and (–)-gallocatechin gallate (GCG). Among these green tea catechins, EGCG has been demonstrated to be bioavailable and could target the molecular pathways in multiple disease models (42–44). It is also the most potent components in terms of biological activities among green tea catechins. The chemical structures of green tea catechins are shown in Figure 2. The chemical structure of polyphenols is characterized by the presence of several hydroxyl groups on different sites of a carbon atom, which may interact with reactive oxidizing species hence inhibiting oxidative stress. Thus, the antioxidant activity of green tea is strongly associated with the electron-rich properties of polyphenols. The 2,3-double bond and the unsaturated 4-oxo group in the C-ring facilitates electron delocalization of o-dihydroxyl catechol within the B-ring (46).

**Figure 3:**
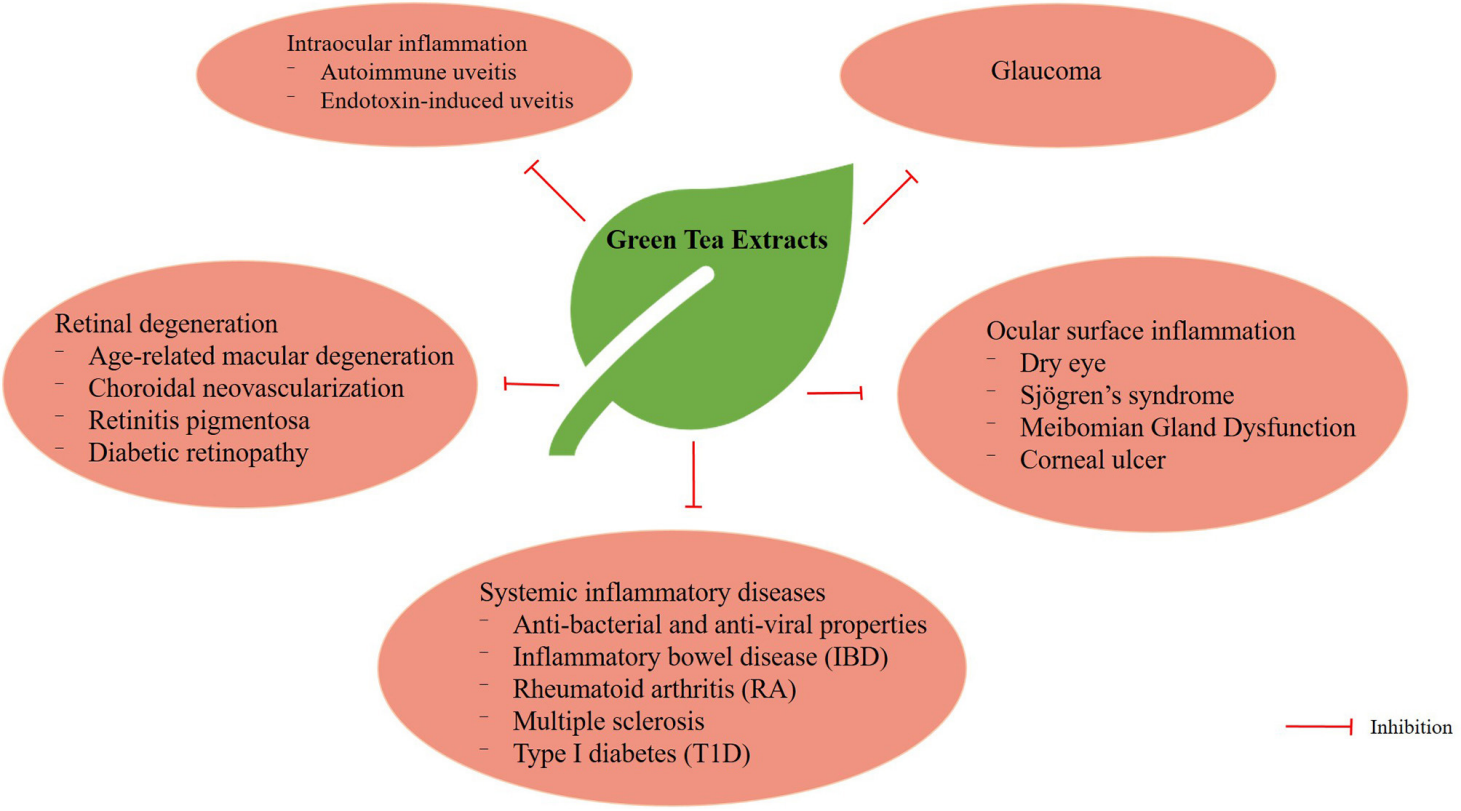
Multiple functions of GTE in various ocular and systemic inflammatory diseases.

The biological activities of catechins are well-known and their antioxidant, anticancer, anticardiovascular, antiobesity and antidiabetic properties have been widely documented (47). In this review, we shall discuss the effects of green tea extracts on autoimmune uveitis, endotoxin-induced uveitis, oxidative-induced retinal degeneration, glaucoma, ocular surface inflammation and systemic inflammatory diseases (Figure 3).

## Pharmacokinetics of Green Tea Catechins in Ocular Tissues

There are few animal studies investigating the uptake and distribution of green tea catechins in ocular tissues and fluids in rats through oral administration. By sacrificing GTE-fed rats at different time intervals to analyze cornea, lens, retina, choroid/sclera, aqueous humor and vitreous humor separately, Chu et al. documented the differential distribution of catechins in various eye tissues (48). GTE was highly concentrated in retina and aqueous humor. The time to reach maximum concentration of GTE after ingestion varied in different tissues ranging from 0.5 to 12.2 h. A further study by the same group revealed that EC, EGC and EGCG were dominant in various ocular tissues except vitreous humor, where GC was predominant (49). In another study of feeding GTE to pregnant rats, catechins could be detectable at higher concentrations in fetal eyes than in other fetal organs including brain, lung, heart, liver, and kidney (50).

## Anti-inflammatory Effects of GTE in Eye Diseases

### Green Tea Catechins in Treating Autoimmune Uveitis

Currently, important findings of numerous basic immunological mechanisms and novel therapies in autoimmune uveitis have been revealed by animal studies based on the mouse models of experimental autoimmune uveoretinitis (EAU) (51–53). Previously, to objectively evaluate the therapeutic effects of anti-inflammatory agents in EAU mice, we developed a novel evaluation system consisting of three quantitative indicators, which are retinal-choroidal thickness (RCT), major retinal vessel diameter and electroretinography (ERG) amplitudes (54). We advocate these live measurements. Objective and more reliable evaluations could be made because the same animals can be followed with the equipment repeatedly. Also less animals are needed for follow-up studies, which make the investigations more ethical. These evaluations are more clinically relevant as similar tests are also regularly performed in human.

We investigated the therapeutic effects of GTE and EGCG in the EAU model (55). After oral administration of GTE, EGCG, dexamethasone, or water, our quantitative evaluation found that GTE, but not EGCG, was able to attenuate several inflammatory signs, including clinical manifestations, histopathological ocular damages, retinal-choroidal edema and retinal vasodilation, in EAU eyes. Therefore, besides EGCG, other catechins and components in GTE may also play roles in EAU alleviation. Interestingly, GTE, as well as high dose EGCG, helped to maintain visual function, which impaired by inflammation in EAU mice. In the EAU model, CD4+ Th17 (IL-17-producing T helper) cells dominantly participated in the inflammation (56). IL-1β and IL-6 are critical factors in determining the lineage choice of differentiating Th17 cells. IL-17A and TNF-α are the hallmark pathogenic cytokines produced by Th17 cells (57). IL-6 also activates CD8+ T cells and inhibits the differentiation of regulatory T (Treg) cells (58, 59). Administration of anti-IL-1β or anti-IL-6 antibody has been shown to attenuate EAU in mice (60, 61) and humans (62). Based on the quantitative results of our studies, GTE and EGCG effectively downregulated the *IL-1*β*, IL-6, IL-17A*, and *TNF-*α expression in retinal tissues from EAU mice, suggesting GTE and EGCG could inhibit EAU by targeting the Th17-associated pro-inflammatory gene expression. Remarkably, GTE treatment showed comparable improvements to the standard dosage of dexamethasone administration in most of the disease phenotypes, or even better in remissions of clinical inflammatory manifestations, vasodilation, and *TNF-*α expression. EGCG showed less effective alleviation of EAU comparing with dexamethasone (55).

### Green Tea Catechins in Treating Endotoxin-Induced Uveitis

Qin et al. investigated anti-inflammatory effects of GTE on endotoxin-induced uveitis (EIU) (63). EIU can be induced by systemic injection of lipopolysaccharide (LPS), mimicking the pathological conditions in human infectious uveitis (64). LPS produced severe hyperemia and edema in the iris. Immunocytochemical examinations showed an accumulation of infiltrating cells in the aqueous humor that were immunopositive for cluster of differentiation 43 (CD43) and CD68, which are markers for leucocytes and macrophages, respectively. Analyses of the aqueous humor showed an increase in pro-inflammatory mediators including tumor necrosis factor-alpha (TNF-a), interleukin-6 (IL-6) and monocyte chemoattractant protein-1 (MCP-1). GTE treatments improved the clinical manifestations and reduced infiltrating cells and protein exudation in the aqueous humor in a dose-dependent manner. The number of CD68 positive macrophages residing in the iris and ciliary was also reduced. GTE suppressed the production of TNF-a, IL-6 and MCP-1 in the aqueous humor, which was associated with a down-regulation of LPS receptor complex subunits, Toll-like receptor 4 (TLR-4) and CD14, and suppression of nuclear factor kappa B p65 (NFkBp65) in the iris and ciliary bodies. Our findings show that GTE is a potent anti-inflammatory agent against the inflammation of EIU, suggesting a potential use in treating acute uveitis.

### Green Tea Catechins in Treating Retinal Degeneration

Several studies have been conducted to explore the anti-oxidative effects of GTE and EGCG in age-related macular degeneration (AMD). Previous studies found that genetic polymorphism in *CFH*, an important regulator of the complement system in innate immunity, is associated with AMD (28, 65). In another study, Zhang et al. induced the oxidative damages in retinal photoreceptors by intraocular injection of sodium nitroprusside (66). Visual function assessed by ERG a- and b-wave amplitudes showed significant reductions after the injection. Decreased expression of photoreceptor-specific markers RET-P1 and rhodopsin kinase and increased cell death marker caspase-3 were demonstrated by RT-PCR and Western blotting after the injection of sodium nitroprusside. Interestingly, EGCG co-injection with sodium nitroprusside significantly blunted these detrimental effects in the retina.

Chronic inflammation could be induced by oxidative stress (67). Yang et al. investigated the anti-oxidative action of GTE in another animal model of oxidative stress specifically to the retina (68). They induced oxidative degeneration of retina in rats by intravenous injection of sodium iodate. *In vivo* imaging by cSLO and SD-OCT observed the pathological changes of the retina, especially in the inner nuclear layer, outer nuclear layer and retinal pigment epithelial layer, caused by sodium iodate. Based on the imaging technology, they found the retinal lesions were significantly ameliorated by GTE. Further analyses of oxidative stress markers revealed reduced expression of superoxide dismutase, glutathione peroxidase, caspase-3 and 8-iso-prostaglandin F2α.

Choroidal neovascularization (CNV) is a key feature in the neovascular subtype of AMD. Laser photocoagulation could be employed to induce CNV in animals. Recently, a study showed that oral feeding of a prodrug of EGCG (pro-EGCG) could significantly reduce the CNV area and vessel leakage in the laser treated mice (69). Mechanistically, pro-EGCG could suppressed the expression of HIF-1α and VEGF, key proteins that can induce the growth of blood vessels, in the inner segment/outer segment (IS/OS) and RPE layers in the retina. Pro-EGCG could also downregulate the proinflammatory M1-type macrophage/microglia polarization and the expression of IL-6 and TNF-α. EGCG has unfavorable bioavailability due to its lower stability in neutral or alkaline conditions in intestines. Also EGCG is hydrophobic which would interfere with its absorption (70). Pro-EGCG can improve the stability and bioavailability of EGCG through acetylation of its reactive hydroxyl groups. Cellular esterase can convert pro-EGCG into EGCG (71). In conclusion, this study showed that EGCG could reduce CNV and suppress inflammation in a wet AMD animal model. In a similar study, light-induced photoreceptor degeneration was alleviated by intraperitoneal injection of EGCG (72). In these animals, cone b wave, and rod a and b waves were all significantly improved after EGCG injection. The expression of antioxidant gene Sod2 was also rescued in EGCG injected animals. Our metabolomic study in human umbilical-vein endothelial cells (HUVECs) found that GTE could exert its anti-angiogenic properties through suppressing synthesis of cellular building blocks and oxidative-phosphorylation metabolites, while promoting the synthesis of membrane lipids and growth factors (73). In another study, polysaccharides purified from GTE was shown to protect human retinal pigment epithelial cells from hydrogen peroxide damage (74). These GTE polysaccharides suppressed the intracellular levels of reactive oxygen species (ROS), as well as elevating antioxidative enzymes including superoxide dismutase (SOD), catalase (CAT), glutathione peroxidase (GPx) and glutathione (GSH). In conclusion, these studies suggesting GTE could modulate ocular neovascularization through multiple pathways.

Retinitis pigmentosa (RP) is a group of heterogenous retinal degeneration diseases caused by various gene mutations that would lead to progression loss of photoceptors. More than 50 genes have been found to be involved in RP (75). One of the widely used animal models, P23H rats, recapitulate the slow rod photoreceptor degeneration in human RP (76). A recent study reported that oral EGCG could help preserve the visual functions in the P23H rats (77). Visual acuity and b wave ERG amplitudes, but not a wave ERG amplitudes, were significantly higher in the EGCG treated P23H rats, compared to the sham treatment group. Interestingly, total antioxidant capacity (TAC) was significantly higher in the EGCG fed P23H rats compared to the sham group. This study demonstrated potential benefits of EGCG in the RP animal model.

Diabetic retinopathy (DR) is a diabetes mellitus (DM) associated irreversible blinding disease that would lead to chronic and progressive changes in the retinal microvasculature. Hyperglycemia in the retinal Müller cells could alter the retinal microvasculature and enhance retinal neuron apoptosis (78). EGCG was reported to induce autophagy, a physiological mechanism to remove cellular components, and protect cells from apoptosis cell death triggered by high glucose treatment in rat Müller cell primary culture (79). In a cross-sectional community study in China, people who consumed tea for more than 20 years was associated with reduced odds of DR (80). The consumption of green tea and non-green tea showed no statistical significance in the DR lower effects. In addition, green tea was found to increase serum antioxidants and to lower the risk of developing type 2 DM (81, 82). In a cellular study, EGCG was found to protect human retinal pigment epithelial cells from pretreatment of methylglyoxal insults, major precursors of advanced glycation end products associated with to DM and DR (83). These studies demonstrated the beneficial effects of GTE in protecting patients against DM and DR.

### Effects of Green Tea Catechins in Experimental Glaucoma Models

Glaucoma is a group of optic neuropathy with progressive retinal ganglion cell (RGC) loss and associated characteristic visual field defects (84). Patients may present in a continuum of severity, ranging from initial asymptomatic stage to late symptomatic stage where there is significant visual impairment from visual field loss and eventually blindness (85). While IOP was identified as the only modifiable risk factor for control of primary open angle glaucoma (86), a certain group of glaucoma patients did not have elevated IOP throughout their course of disease, and the mechanism for normotension glaucoma is not completely clear. In recent years, mounting evidence showed that neuroinflammatory processes, immune-relevant responses and oxidation play key roles in the pathogenesis of glaucoma (13, 14). A number of molecular pathways have been suggested to regulate inflammation in human and animal glaucoma models (15). For examples, Toll-like receptor related pathway (87), TNF-α pathway and a related protein FasL (88, 89), and the complement cascade (90, 91) were involved in experimental glaucoma or in RGC death. Besides, various pro- and anti-inflammatory cytokines such as interleukins (ILs) and interferons (IFNs) are suggested to involve in glaucoma during antigen presentation to T cells (15, 92, 93). Although there were conflicting results, a meta-analysis suggested that flavonoids may have a role in improving visual function in patients with glaucoma and ocular hypertension (94). Our recent study found that GTE could alleviate RGC degeneration in ischemic reperfusion rats (95). Apoptosis, oxidative stress and inflammation were suppressed in RGC by the GTE treatment. One major function of RGC, the pupillary light reflex, was also significantly rescued by GTE. This study suggested GTE could be developed as a novel treatment for glaucoma and other optic neuropathies. In another mechanistic study, EGCG was found to stabilize the growth cone at the tip of growing *Xenopus* RGC by stabilizing F-actin within the growth cone (96). These findings demonstrated EGCG could modulate RGC guidance, which could be further investigated its potential in glaucoma treatment. In an experimental glaucoma mouse model, injection of microbeads in the anterior chamber was able to block the aqueous outflow and elevate IOP. The resulting RGC loss, but not IOP elevation, could be reversed by oral EGCG (97). This study demonstrated EGCG could directly protect RGC, which may be developed as an alternative treatment to glaucoma in addition to the existing clinical practice of controlling elevated IOP in glaucoma patients. Indeed, a randomized controlled study on the effect of short-term use of epigallocatechin-gallate (EGCG), a powerful antioxidant, in ocular hypertension and open-angle glaucoma patients suggested that EGCG had a small but favorable effect on the inner retinal function as assessed by pattern electroretinogram in eyes with early to moderately advanced glaucomatous damages (98). These experimental results suggest the beneficial effects of green tea catechins in glaucoma. More large scale and long term studies are needed to evaluate the efficacy of green tea catechins in treating glaucoma.

### Green Tea Catechins and Ocular Surface Inflammation

Both infectious and autoimmune etiologies are involved in the inflammatory process on ocular surface. As one of the most common ophthalmic pathologies, dry eye disease affects millions of people in developed regions (99). It has been recognized that dry eye is associated with inflammation of ocular surface, especially the Th1 (100) and Th17 (101) pathways. Numerous inflammatory cytokines and chemokines such as IFN-γ, TNF-α, IL-1β, IL-6, IL-17, C-C motif ligand 2 (CCL2) and matrix metalloproteinases (MMPs) have been implicated in dry eye (102–104). Several studies have suggested that green tea catechins, especially EGCG, which displays anti-inflammatory properties, are beneficial to the health of ocular surface. Administration of the green tea catechins protected against autoimmune-induced pathological changes in the salivary glands in animal models suffering Sjögren's syndrome (105) and helped to inhibit dry eye disease-related inflammation (106). Cellular studies also demonstrated that EGCG inhibited release of IL-6, IL-8, monocyte chemotactic protein-1 (MCP-1), granulocyte-macrophage colony-stimulating factor (GM-CSF), and granulocyte colony-stimulating factor (G-CSF) in human corneal epithelial cells, implying a therapeutic role for ocular surface inflammatory conditions (107). Moreover, a double-blind randomized controlled clinical trial with 60 patients of dry eye and Meibomian Gland Dysfunction (MGD) showed that GTE improved the clinical symptoms, tear breakup time (TBUT), and the health of meibomian glands. No side effect of the treatment was observed (108).

To study corneal ulcer, proinflammatory cytokine IL-1β is able to induced corneal collagen degradation (109). In IL-1β treated three-dimensional (3D) cultured human corneal fibroblast, EGCG treatment could suppress collagen degradation in a concentration-dependent manner (110). EGCG was found to suppressed the conversion of exogenous plasminogen to plasmin induced by IL-1β, leading to the inactivation of pro-MMP1. The lower matrix metalloproteinase activities resulted in less collagen degradation. This study highlighted the potential of EGCG is treating corneal ulcer.

## Effects of Green Tea and Catechins on Other Systemic Inflammatory Diseases

Some ocular inflammation can be caused by systemic inflammatory diseases including ankylosing spondylitis, Behçet disease, and Vogt–Koyanagi–Harada disease. Therefore, green tea and catechins could help to treat ocular inflammation by resolving relevant systemic inflammatory diseases. Taking a view outside the eye, green tea catechins also possess anti-inflammatory, antioxidative, antiproliferative, and antibacterial, and antiviral properties by binding to the corresponding biological molecules and regulating a variety of enzyme activities and signal transduction pathways (111). In 1985, Dr. Tsuneo Kada of National Institute of Genetics of Japan reported that bacterial mutagenicity could be suppressed by green tea brew (112). This had led to subsequent studies to investigate the protective effects of GTE against infections. Later, the anti-oxidative effect of GTE was identified (113). Physiologically, GTE possesses the anti-bacterial and anti-viral properties against foodborne pathogenic bacteria, dental caries bacteria, *Helicobacter pylori*, Methicillin-resistant *Staphylococcus aureus* (MRSA), influenza virus, human papilloma virus, and herpes virus (114).

Green tea consumption has also shown protections against autoimmune diseases in experimental models. The effects are suggested to be owing to its high content of polyphenols. EGCG is the most abundant molecule among all catechins. Studies in animals showed that green tea catechins or EGCG can resolve autoimmune disorders such as inflammatory bowel disease (IBD) (115, 116), rheumatoid arthritis (RA) (117–120), multiple sclerosis (121), type I diabetes (T1D) (122), and Sjögren's syndrome (105, 123). In these studies, researchers consistently observed that administration of GTE or EGCG was able to alleviate these diseases by decreasing the disease incidence and severity, improving the clinical manifestations, reducing pathological damages and serum/tissue autoantibody levels, and suppressing relevant pro-inflammatory gene expression. Although it has been reported that EGCG acts as an anti-inflammatory agent by suppressing CD4+ T cells (124), more studies are still needed to investigate the protective mechanisms of GTE in autoimmune diseases. Animal studies in experimental autoimmune encephalomyelitis revealed that EGCG attenuates the autoimmune inflammation by suppressing the proliferation of T cells and the production of pro-inflammatory cytokines, decreasing Th1 and Th17 populations and increasing Tregs in lymph nodes, spleen and the central nervous system (CNS) (125).

## Safety of Green Tea Consumption and GTE Administration

For centuries green tea has been extensively consumed as a daily beverage in Asia-pacific region such as China, Japan and India (126, 127). It has been regarded as a safe agent to human. Although green tea as a beverage is not equal to GTE, many patient-based studies did not identify any serious adverse effects by using low to moderate doses of GTE or its most biologically potent polyphenol constituent, EGCG (111, 128, 129). However, GTE could possibly be contaminated by toxic elements, heavy metals and pesticides (130). In addition, there are reported studies showing adverse effects related to high dose of green tea consumptions (10 to 29 mg/kg/d) (131, 132). Most of these adverse effects were hepatitis. Serum alanine aminotransferase (ALT) and bilirubin levels were elevated (131, 133). And these adverse effects could be resolved spontaneously following the termination of green tea consumption. Histological verification of the liver indicated inflammatory reactions, cholestasis, occasional steatosis and necrosis. The adverse effects were probably associated with the intra-individual differences in the metabolism of green tea catechins. Excessive oxidative stress may also contribute to the adverse effects in the liver (134). Laboratory studies of GTE in animals have also revealed toxicity under high doses usage. Isbrucker et al. applied the catechins to rats at a dose of 2,000 mg/kg resulting in 80% of the mortality. Hemorrhage in the stomach and intestine was observed. These data implied that high doses of EGCG could induce toxicity in the liver, kidney and intestine (135). Study in mice by Lambert et al. also demonstrated that oral administration of high doses of EGCG (1,500 mg/kg or 750 mg/kg) induced hepatotoxicity which was caused by the oxidative stress in hepatocytes. A 5-fold increase of hepatic lipid peroxidation, 9.5-fold increase of plasma 8-isoprostane level and increased expression of hepatic metallothionein were observed (136). However, these adverse effects inducing doses are much higher than the regular dietary consumption of green tea and the minimal effective doses suggested by previous studies.

The oral bioavailability of green tea catechins in human is low (137). The plasma concentrations of green tea catechins after ordinary amount of green tea consumption in human were 5-50 times less than the concentrations in *in vitro* studies (138–140). Many environmental and gastrointestinal conditions including temperature, acidity and other biological enzymes have been reported to affect the stability of GTE (141, 142). Nanotechnology has been one of the major research directions to improve the stability of GTE (143). Encapsulating green tea catechins with nanoparticles including liposome and other liposomal-like structures phytosomes and niosomes have been reported to improve the antioxidant activity and chemical stability of EGCG (144). Nanocarriers made of polysaccharides including chitosan, cyclodextrins and alginate could protect catechins from degradation under a range of temperatures and pH conditions (145). Proteins including bovine serum albumin, gelatin and β-lactoglobulin could protect EGCG against oxidative degradations and lessen the bitter taste of EGCG (143). In terms of safety concerns of encapsulated green tea catechins, no cytotoxic effects were observed in the positive surface charge chitosan at concentration rang of 0.002–0.5 mg/ml (146). EGCG encapsulated in casein micelles demonstrated cytotoxicity to cancer cells but not in non-cancer normal cells at concentrations <0.15 mg/ml (147). These studies suggested encapsulation could enhance the bioavailability of green tea catechins efficiently.

## Future Perspective and Research Opportunities

Asian and Mediterranean diets consisted of difference herbal molecules are gradually being appreciated due to their health benefits, including protective effects against neurodegenerative and cardiovascular diseases (148). Natural components that are extracted from diet have offered further insights to explore novel therapeutic strategies for challenging diseases such as intraocular inflammation. GTE, one of the agents possessing potent anti-inflammatory property, is promising for prevention and treatment of intraocular inflammation. Recent epidemiological findings show that green tea consumption is able to decrease the risk of cognitive dysfunction (149, 150). It is becoming clear that the potent anti-inflammatory effects of GTE on the eye involve both infectious and autoimmune pathogenesis. For future perspective, from the studies of autoimmune uveitis, although EGCG is the most abundant constituent of GTE, the efficacy of GTE in treating EAU mice could not be fully replicated by EGCG (55). Therefore, in the future, other green tea catechins and non-catechins molecules should also be studied individually and in combinations to find out the best formulae for treating autoimmune uveitis. In addition, these formulae can also be studied in combination with steroids, which may allow us to achieve better treatment outcomes.

Human beings are sharing many evolutionarily conserved mechanisms with other species which could have been inherited during evolution. However, the findings of these experimental models cannot be directly applied to human, due to our complicated physiological and cultural conditions, which cannot be observed in the animal models. Clinical investigations are still warranted for translational studies from bench to bedside. Similarly, in the case of GTE, the efficiency and safety studies provide a novel strategy for inhibiting disease progression in intraocular inflammation, which we have discussed in this review. Future studies should focus on patients. Clinical trials could be organized to clarify the optimum dose of GTE in human to achieve the maximum health benefits and minimum side effects.

## Author Contributions

All authors participated in the literature review and manuscript writing.

## Conflict of Interest

The authors declare that the research was conducted in the absence of any commercial or financial relationships that could be construed as a potential conflict of interest.

## Publisher's Note

All claims expressed in this article are solely those of the authors and do not necessarily represent those of their affiliated organizations, or those of the publisher, the editors and the reviewers. Any product that may be evaluated in this article, or claim that may be made by its manufacturer, is not guaranteed or endorsed by the publisher.
